# Mental Rotation Performance: Contribution of Item Features to Difficulties and Functional Adaptation

**DOI:** 10.3390/jintelligence13010002

**Published:** 2024-12-30

**Authors:** Mehdi Rajeb, Andrew T. Krist, Qingzhou Shi, Daniel O. Oyeniran, Stefanie A. Wind, Joni M. Lakin

**Affiliations:** 1 Department of Educational Studies in Psychology, Research Methodology, and Counseling, College of Education, The University of Alabama, Tuscaloosa, AL 35487, USA; atkrist@crimson.ua.edu (A.T.K.); dooyeniran@crimson.ua.edu (D.O.O.); stefanie.wind@ua.edu (S.A.W.); jlakin@ua.edu (J.M.L.); 2 Department of Psychology, Northwestern University, Evanston, IL 60208, USA; qzshi46@hotmail.com

**Keywords:** mental rotation test, spatial ability, LLTM, item characteristics, Rasch model

## Abstract

Mental rotation is an important aspect of spatial ability. While the importance of measuring mental rotation has been explored, disputes still exist within the literature surrounding sources of item difficulty in mental rotation tests (MRTs). Furthermore, gender differences in MRT performance are often seen but not fully understood. In the current study, we analyzed sources of item difficulty in a set of spatial ability test items using the Linear Logistic Test Model (LLTM). We found that items with more cubes, color differences, and higher rotational complexity tend to be more difficult, whereas items that contain occlusion, a mirrored structure, and a homogenous configuration type tend to be easier. Next, using Differential Component Functioning (DCF) analysis, we analyzed gender differences across these different item characteristics, finding that the number of cubes and color characteristics made questions more difficult for males when compared to females. The results and implications of this study are discussed in further detail.

## 1. Introduction

Spatial ability, or “the ability to generate, retain, retrieve, and transform well-structured visual images” (Lohman 1994, p. 3), is a highly influential yet often overlooked area of cognitive processing in STEM education. Individual differences in spatial ability are not tested at the same rate as differences in mathematics and language ability (Anderson 2014; Kell and Lubinski 2013). Because of this, people who have high spatial ability often underachieve when compared to people who are talented in other abilities (Gohm et al. 1998; Lakin and Wai 2020). To combat this, research must focus on developing high-quality spatial tests that can identify spatially talented individuals at an early age (Shea et al. 2001; Webb et al. 2007).

Spatial ability can be broken down in various ways to specific skills. One predominant structure suggests that the strongest factors are as follows: (1) spatial visualization, which is the ability to encode spatial information and reason about those representations, (2) spatial orientation, including the ability to imagine a scene from different perspectives, and (3) mental rotation (Carroll 1993; Casey 2013). Mental rotation is often classified as a dynamic spatial ability achieved through an intrinsic cognitive process (Uttal et al. 2013). Mental rotation ability has been shown to be a key indicator of mathematics ability (Bruce and Hawes 2015; Lowrie et al. 2016; Zhao et al. 2021). Because of this, mental rotation is often incorporated into scales measuring spatial ability, exemplified by tests such as the Purdue Spatial Visualization Test (PSVT; Guay 1976), adaptations of the PSVT (Branoff 2000), the Vandenberg and Kuse Test (Vandenberg and Kuse 1978), and the Spatial Reasoning Instrument (Ramful et al. 2017).

In an investigation by Jost and Jansen (2022) that revisited earlier research (e.g., Heil and Rolke 2002; Just and Carpenter 1985; Shepard and Cooper 1986), it was established that people navigate through distinct stages while performing mental rotation tasks. These stages encompass the following: (1) stage of perceptual processing (i.e., perceptual identification and visualization of the object), (2) stage of performing mental rotation itself, and (3) the stage of decision-making culminating in the selection of final response. Additionally, Lohman (1988) inferred that high abilities in visualization, identifying orientation of an object or its rotation, represents elevated dimensions of spatial skills. Carroll (1993) proposed that executing spatial tasks requires abilities for the visual apprehension of an object’s forms and shapes. Consequently, the perceptual processing of objects involved in mental rotation tasks assumes a significant role in explaining individual performance in mental rotation tests (MRTs).

Differences in both individual performance and item difficulty in MRTs have been recorded (Bartlett and Camba 2023b; Caissie et al. 2009). Buckley et al. (2018) found that when solving MRT items, paradoxically, effort decreases as item difficulty increases. Furthermore, people tend to be less efficient at encoding target stimuli as task difficulty increases (Snowden et al. 2021). This lack of efficiency and effort in more difficult mental rotation items may be due to a lack of familiarity that people have with these types of items (Buckley et al. 2018). Branoff (2000) found that the typical isometric orientation of MRT items, where the X, Y, Z axes are all 120° from each other, was confusing to students who were not used to that type of representation. They suggested that a trimetric orientation for items, where the angles are unequal and the perspective is more naturalistic, can be less confusing than isometric items due to the lack of context that is given in isometric figures.

Multiple studies have been conducted investigating the sources of difficulty in MRT items and have produced mixed results. It is accepted that two-dimensional MRT items tend to be easier than three-dimensional items (Ramful et al. 2017). This is due to the extra rotational dimension that is present in 3D items, as well as the possibility of occlusion that is not present in 2D items (Jolicoeur 1985). According to Caissie et al. (2009, p. 95), occlusion is present when “significant parts of the three-dimensional figure are covered” by other parts of the same figure. In their analysis of MRT item difficulties, Caissie et al. (2009) put forth that diverse item attributes such as occlusion, complexity (mirror images vs. non-mirror images), and configuration type (homogenous vs. heterogenous) account for a substantial portion of the variation in item difficulty. This contrasted with the findings in an earlier study by Cooper (1975), who stated that the angle of orientation contributed to item difficulty more than shape complexity. Furthermore, Caissie et al. (2009) disputed the findings in Bejar (1986), who stated that angular disparity (like the “angle of orientation” discussed in Cooper (1975) significantly affects item difficulty. Takahashi and Connolly (2012) suggested that the perceptual processing in an MRT task (i.e., comprehending or encoding the shape) should be treated as a distinct spatial task, heavily contingent on item attributes. In summary, it can be inferred that item attributes, such as visual characteristics and complexities, play a pivotal role in explaining the perceptual processing of an MRT task, subsequently influencing MRT item difficulties and individual test performances. However, the disagreements found in the literature surrounding the specific sources of item difficulty leave room for further investigation.

A further consideration in mental rotation is the magnitude of gender difference, which may play a role in explaining gender differences in STEM career pursuit (Halpern et al. 2007; Wang and Degol 2017). Gender differences in MRT scores are among the largest found in the literature (Reilly and Neumann 2013; Voyer et al. 1995). However, while these differences are apparent, it is not obvious why these differences exist. Arrighi and Hausmann (2022) found that anxiety and self-confidence are mediators for performance differences across genders in MRT items, especially in items with high difficulty. Some studies have investigated the differences in strategy use across genders. Hirnstein et al. (2009) found that while men use more of a leaping strategy in their problem-solving (i.e., rapidly choosing a response without reviewing all options), this does not significantly explain the performance differences across gender. Toth and Campbell (2019) investigated gender differences in computerized MRT in both performance and strategy use but found no significant differences between genders.

While these studies attempt to explain gender differences in construct-relevant terms, Bartlett and Camba (2023a) highlight that the MRT was intentionally designed to maximize gender differences in performance, thereby reinforcing stereotypes of male superiority in spatial ability. They contend that the MRT’s gender biases and reliance on construct-irrelevant factors render it an unreliable measure of true spatial ability. Addressing the conflicting explanations for gender differences in MRT performance remains a critical area for future research.

Efforts to design more equitable assessments of mental rotation abilities have increasingly focused on the influence of stimulus features and their potential to bias performance across genders. For instance, Ruthsatz et al. (2015) demonstrated that using gender-stereotyped items, such as cars versus dolls, can significantly affect children’s mental rotation performance, with stereotypically feminine items reducing the gender gap. Similarly, Ruthsatz et al. (2014) explored how the use of alternative figures, such as pellet figures instead of cubes, influence task difficulty and equity in performance among boys and girls. These findings underscore the importance of item selection and design in minimizing unintended biases in mental rotation tasks. Bartlett et al. (2024) introduced a virtual reality mental rotation test as a novel approach to assessing spatial abilities, highlighting how technology can provide more immersive and equitable testing environments. These studies are a few examples of research that has helped address the lack of valid and inclusive spatial assessment tools outlined in Uttal et al. (2024).

Our study builds on this body of work by examining how various item characteristics, including color, configuration, and structure, impact performance difficulty. By incorporating different features and evaluating their effects, we aim to contribute to a more inclusive and equitable assessment of mental rotation abilities. This approach aligns with the broader goal of developing tasks that are accessible and fair for individuals with diverse experiences and skill sets.

### Research Objectives

**Figure 1:**
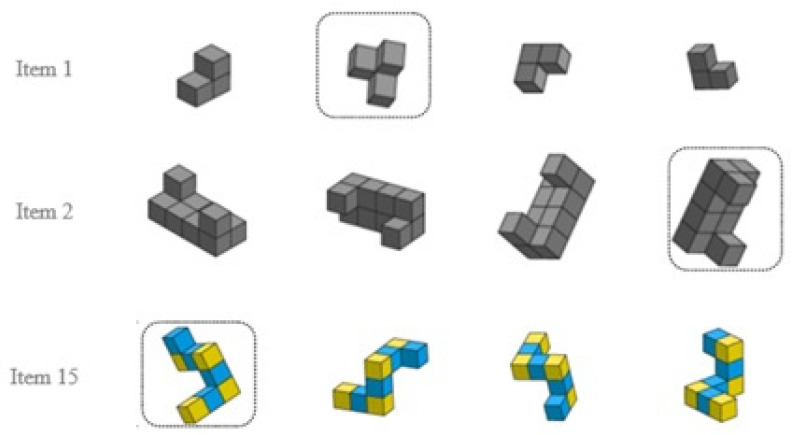
Example of mental rotation task. Examinees were asked to identify the object that is a different shape from the other three. Answers are identified by a rectangular box.

Performance in MRT shows considerable variation within and across individuals, implying a corresponding variability in item difficulties (Bartlett and Camba 2023b; Caissie et al. 2009); see Figure 1. Certain item attributes, such as the degree of rotation, hidden features of the shape, and other visual characteristics, have been shown to influence item difficulty (Caissie et al. 2009; Takahashi and Connolly 2012). Also, gender differences have been observed in MRT performance (Hirnstein et al. 2009; Taragin et al. 2019). However, these differences are complex and may be due to external factors (Arrighi and Hausmann 2022). Hence, this study aims to explore the following research questions revolving around MRT items:

(1) To what extent do item-related attributes explain variation in difficulty for MRT items?

(2) To what extent do the effects of item attributes on difficulty vary by gender?

## 2. Materials and Methods

### 2.1. Participants

This research was undertaken within the scope of a broader initiative aimed at creating a spatial reasoning assessment for students in 3rd to 8th grade. Students’ demographic distributions are presented in Table 1. Students in grades 3 to 8 (or rising to that grade in the next school year) were recruited through summer camp programs held at one of two university programs (one in the southeast, the other in the Midwest regions in USA). The camp programs focused on a range of academic, artistic, and STEM-related topics. Parents or guardians of students were contacted via email to have their students participate in the research study. Parents registering their children for this study were asked to provide their child’s age and gender as part of the data collection process. Students received a gift card for their participation.

### 2.2. Instrument

The research team developed 15 mental rotation questions, categorized by Eliot and Smith (1983) as a member of block rotation tests. These items were developed based on the multiple-choice variation of the mental rotation test of three-dimensional spatial visualization proposed by Vandenberg and Kuse (1978; see Hegarty 2018). Unlike the MRT proposed by Vandenberg and Kuse’s (1978) true–false task, where all figures have the same number of blocks and follow a consistent structure, the MRT employed in this study features shapes that differ in block count or configuration. These variations on the original test are widely used (Uttal et al. 2024).

We developed items with a range of item features (number of cubes, degree or rotation, occlusion) to impose potential variabilities in item difficulties. These items were pilot-tested previously with several hundred young students and college students to ensure item quality and internal consistency of the scale. The item features considered in this study were not included within each item as part of the assessment design. Instead, the research team explicitly identified item characteristics embedded in each of the MRT items after the items were administered. A few sample items and item characteristics considered in this study for the MRT are presented in Figure 1 and Table 2.

### 2.3. Data Analysis

#### 2.3.1. Exploration of Psychometric Properties of MRT

The MRT items were analyzed in multiple steps to answer the research questions. To investigate internal consistency of MRT items, we obtained corrected item-total correlations (Henrysson 1963) and Cronbach’s alpha (Cronbach 1951). The corrected item-total correlation represents the correlation between the individual item and the overall score when that specific item is excluded from the instrument, whereas Cronbach’s alpha is a statistical measure for internal consistency. Moreover, summary statistics for item responses were obtained for each item so that we could explore the overall item and response characteristics. For this analysis, the psych package (Revelle 2023) in R version 4.2.1 (R Core Team 2022) was used.

After investigating the overall psychometric properties of the MRT instrument, we used the explanatory Item Response Theory (IRT) framework (De Boeck and Wilson 2004) to estimate the effects of item features on item difficulties. Explanatory IRT models are well suited to explore how item characteristics influence item difficulties (Isbell and Son 2023). To address the research questions of the current study, we employed the Linear Logistic Test Model (LLTM) for dichotomous responses. The LLTM is an item-explanatory IRT model and an extension of the Rasch model (Embretson and Reise 2000). As emphasized by Green and Smith (1987), it is essential to ensure adequate psychometric properties of the MRT item response data and item fit evidence prior to interpreting the estimates from extended IRT models. Hence, Rasch analysis followed by LLTM analysis was performed.

#### 2.3.2. Rasch Analysis

After exploring the overall psychometric properties of MRT, the dichotomous Rasch model (Rasch 1960) was fitted to the MRT item response data. In keeping with standard procedures for evaluating fit to the Rasch model, we performed several analyses. First, we examined the overall model fit for the Rasch model by employing a likelihood ratio test. Second, to assess unidimensionality of the MRT test, we used a principal component analysis (PCA) of standardized residuals (Chou and Wang 2010; Raîche 2005; Wind and Schumacker 2021) with a recommended threshold eigenvalue of 2.0 for satisfactory unidimensionality (Linacre 2009). Next, we examined whether items in MRT are statistically independent by investigating the inter-item residual correlation after accounting for their correlation with the latent construct being measured. Finally, we examined item-level indicators of fit using two types of statistics: (1) the residual summary statistics (infit and outfit mean square error [*MSE*]) and (2) an analysis of Differential Item Functioning (DIF) for individual items.

*Infit* and *outfit* mean *MSE* fit statistics are item fit statistics commonly used in the context of Rasch models to examine the fit of individual items and individual people (Linacre 2002). These statistics are essential for identifying anomalous responses and help us identify the extent to which item response patterns adhere to Rasch model requirements. In general, researchers agree that these statistics are expected to have a value of 1 when there is acceptable fit to a Rasch model, with values exceeding 1 indicating frequent or substantial unexpected responses and values lower than 1 indicating less variation in responses, which may be attributable to inter-item dependency or response effects (e.g., carelessness). Hence, *infit* and *outfit MSE* statistics were obtained for each person and each item in the MRT test and compared with common critical values.

#### 2.3.3. Differential Item Functioning for Gender

We used Differential Item Functioning (DIF) analysis to assess the degree to which MRT items have invariant difficulty levels across gender groups. DIF is observed when individuals from different groups (e.g., gender, age group) who are otherwise matched on ability level do not have a similar likelihood to answer the item correctly. As suggested by Freitas et al. (2014), the existence of DIF can negatively affect the validity of scores by introducing construct-irrelevant variance. This occurs when external factors unrelated to the measured attribute influence item response, compromising the validity of the score interpretations. In this study, we explored whether any item in the MRT instrument showed evidence of potential DIF related to participant gender (male and female). To perform the DIF analysis, the dichotomous Rasch model was employed to estimate item difficulty and corresponding standard error for each group (i.e., male and female). Furthermore, following Wright and Masters (1982), the standardized differences in item difficulty between male and female students are obtained by

(1) $$\;z=\frac{\left(d_{m}-d_{f}\right)}{\sqrt{se_{m}^{2}+se_{f}^{2}}}$$

Here, z is the standardized difference, $d_{m}$ is the item difficulty for items in the male subgroup, $d_{f}$ is the item difficulty for items in the female subgroup, $se_{m}^{2}$ represents standard error for $d_{m}$, and $se_{f}^{2}$ represents standard error for $d_{f}$. A high z value indicates that an item is more difficult for males compared to females and vice versa. Furthermore, the z value is tested against the standardized normal z values at a 0.05 level of significance to obtain the statistical significance of the differences in item location parameters for different groups. In addition, the logit differences in item difficulties are obtained to compare how item difficulties vary across genders. To perform the DIF analysis, the extended Rasch model (eRm; Mair et al. 2021) R-package was used.

#### 2.3.4. Analysis Employing Linear Logistic Test Model

In addition, to assess the impact of various item features on the difficulty of an item, we employed an extended IRT model, the Linear Logistic Test Model (LLTM; Fischer 1995). LLTM has been employed in several other similar studies to examine item characteristics (e.g., Hartig et al. 2012; Krell et al. 2021). The LLTM is an extension of the Rasch model (De Boeck et al. 2016). To explain the LLTM, let us specify the dichotomous Rasch model as

(2) $$\;P\left(Y_{ni}=1\right)=\frac{e^{\theta_{n}-\delta_{i}}}{1+e^{\theta_{n}-\delta_{i}}},\;$$

where $P\left(Y_{ni}=1\right)$ is the probability that *n*-th examinee responded to item *i* accurately, $\theta_{n}$ represents the person ability parameter for *n*-th examinee, and $\delta_{i}$ represents the difficulty parameter for item *i*. For LLTM, let us assume that $\delta_{i}^{\prime }$ and $\theta_{n}^{\prime }$ are estimated item difficulty parameters and person’s ability parameters, respectively. The LLTM is explanatory in nature as it assumes that the item difficulty parameter can be explained by a linear combination of basic parameters (Krell et al. 2021). In general, the $\delta_{i}$ parameters of the Rasch model are replaced with a linear combination of the basic parameters, i.e.,

(3) $$\;\delta_{i}^{\prime }=\overset{J}{\underset{j=1}{\sum }}q_{ij}\eta_{j},$$

where $q_{ij}$ is the assigned weight of basic parameter *j* for item *i*, and $\eta_{j}$ denotes the estimated difficulty contributed by the basic parameter *j.* In other words, as explained by Krell et al. (2021), $\eta_{j}$ is the regression coefficient for *j*-th basic parameter, i.e., the estimated contribution of item feature *j* to the overall item difficulty. To estimate the LLTM model, we employed the Q-matrix presented in Table 3. The Q-matrix was developed based on expert opinions on MRT items as well as previous studies conducted surrounding this topic. Three members of the research team coded the items according to characteristics defined in the Q-matrix; any discrepancies were discussed and resolved prior to analysis.

In the Q-matrix, a total of six item characteristics were considered. One polytomous item characteristic, the number of cubes, was dichotomized to ensure model fit and interpretability. To dichotomize this characteristic, we used a mean-split to divide the items into two groups. The mean number of cubes for all the items was 8.6. We dichotomized the number of cubes considering 8.6 as a threshold point, i.e., if any item possesses more than 8.6 cubes then we assigned 1 for that item in the Q-matrix, otherwise 0. All other item characteristics were defined as dichotomous characteristics in the Q-matrix (i.e., 1 if the item possesses the characteristics or 0 otherwise). For instance, the color is specified as 1 in the Q-matrix if the distractor of any item contains multiple colors or 0 otherwise. Furthermore, image structure is composed of two characteristics, i.e., structural image and mirror image. We specified image structure as 0 if the item distractors are structurally different or 1 if they are mirrored images of the original item. Similarly, the item configuration characteristic is divided into two segments, i.e., homogenous and heterogeneous. In this study, we assumed that homogenous items contribute to higher levels of difficulty, and hence, in the Q-matrix, we specified homogenous items as 1 and heterogeneous items as 0 (see Table 2 for more details).

As suggested by Baghaei and Ravand (2015), we assessed the adequacy of the model–data fit of LLTM in multiple ways. First, we assessed whether the Rasch model fit the MRT item response data adequately. Fischer (1973) suggested that prior to applying LLTM, the item response data should fit the Rasch model. Second, we employed the log-likelihood difference test to compare the model–data fit of both the Rasch model and LLTM (Krell et al. 2021). Specifically, we computed Pearson correlation coefficients between the item difficulty parameters derived from the Rasch model and LLTM. The correlation coefficient allows us to explore the extent to which the LLTM’s item characteristics contribute to the variance in item difficulty estimated by the dichotomous Rasch model. A strong correlation between the set of estimated difficulty parameters from the Rasch model and LLTM suggests the validity of the decomposition of difficulty parameters (Fischer 1973; Krell et al. 2021).

#### 2.3.5. Differential Component Functioning (DCF) for Gender via LLTM

To explore how different item characteristics contribute to item difficulties for students across genders, this study uses Differential Component Functioning (DCF). DCF is an adaptation of DIF in which we used separate LLTM calibrations for male and female students and compared component difficulty estimates between groups. To perform the DCF, we first divided the sample into two groups and fitted the LLTM model separately for male and female responses. We then compared the effects of item characteristics on item difficulty across gender. Furthermore, we attempted to explore which item characteristics significantly differ across gender. To explore differences in the effects of item characteristics between groups, we performed a post-estimation test for each item characteristic. To perform the post-estimation test, we employed Wald’s Chi-square test proposed by Liao (2004), which is presented below.

(4) $$W=\frac{{\left(\eta_{male}-\eta_{female}\right)}^{2}}{SE{\left(\eta_{male}\right)}^{2}+SE{\left(\eta_{female}\right)}^{2}}\;\sim \;\chi_{1}^{2}$$

Here, $\eta_{male}$ and $\eta_{female}$ represent the effects of certain item characteristics on item difficulties for male and female students, respectively, and *SE* represents standard error for the estimated parameters. For group-wise effect comparisons, Wald tests are considered robust tests (Liao 2004; Stansfield and Parker 2013). Several other studies have employed the Wald test for comparing effects from group-wise estimated models (e.g., Ingram and Hinduja 2008; Ireland and Smith 2009; Pratt et al. 2022). Since the Wald test is performed in a post hoc setup and effects of multiple item characteristics are compared simultaneously, we adopted Bonferroni correction to adjust the Type I (i.e., α) error rate (Armstrong 2014) when deciding the difference in effects of item characteristics. Hence, we considered the Bonferroni-corrected level of significance, α*=αT, for *T* number of statistical tests (Shi et al. 2012).

## 3. Results

### 3.1. Psychometric Properties of MRT Items

Preliminary psychometric analysis of MRT items displayed acceptable internal consistency; summary statistics are presented in Table 4. Most of the items in the MRT instrument demonstrate moderate difficulty levels based on proportion-correct statistics. Among all 15 items, item 1 (*M* = 0.98) and item 11 (*M* = 0.92) were relatively easy, while item 9 (*M* = 0.24), item 15 (*M* = 0.29), and item 7 (*M* = 0.30) were relatively difficult. Furthermore, the majority of the corrected item-total correlations for MRT items were positive and within the acceptable range of 0.30 to 0.70, as suggested by Ferketich (1991). Cronbach’s alpha for the MRT is 0.79, which surpasses the often recommended critical value of 0.70 (Nunnally 1978).

### 3.2. Results from the Dichotomous Rasch Model

To explore the model–data fit of the dichotomous Rasch model, the likelihood ratio (LR) test was performed, and the resulting test measures were $\chi^{2}\left(12\right)=20.37,\;p>0.05$, suggesting that the MRT item response met the model requirements adequately. The Rasch estimates (of examinee and item locations) explained 39.2% of the variation in MRT item responses, which approximates a large effect size, as suggested by Cohen (1992). Moreover, eigenvalues from a principal component analysis of standardized residuals were ≤1.89, indicating that the MRT item responses are sufficiently unidimensional for practical interpretation purposes (Chou and Wang 2010). Near-zero correlations (*M* = −0.06, *SD* = 0.097) were observed among residuals associated with each item, suggesting local independence in MRT items. In addition, the summary measures for item fit statistics (infit *MSE*: *M* = 0.96, *SD* = 0.16; outfit *MSE*: *M* = 0.93, *SD* = 0.34) and the summary measures for person fit statistics (infit *MSE*: *M* = 0.97, *SD* = 0.33; outfit *MSE*: *M* = 0.93, *SD* = 0.79) suggested that, overall, the estimated item and person parameters met the criteria for adequate model–data fit for Rasch model (Hohensinn and Kubinger 2011; Smith 2004).

Table 5 presents item calibration for all items used in the MRT including the estimates for difficulty parameters, standard errors, and relevant item fit statistics. In summary, the results of the dichotomous Rasch model analysis indicated that the LLTM can be applied to explain item difficulties based on item characteristics. The item difficulty parameters estimated from MRT item responses range from −3.92 logits (*SE* = 0.56 for item 1) to 1.83 logits (*SE* = 0.24 for item 9). The variation in difficulty parameters for different items suggests that items in MRT required different levels of ability to correctly respond to MRT items. We further explored the item fit statistics obtained from the Rasch analysis for MRT data. The item fit statistics indicated that the MRT items adequately fit the dichotomous Rasch model for most items. The largest fit statistics were obtained for item 9 (outfit *MSE* = 1.58; infit *MSE* = 1.04), and the smallest fit statistics were obtained for item 1 (outfit *MSE* = 0.32; infit *MSE* = 0.79). However, standardized fit measures for item 5 (standardized outfit *MSE* = 2.30; standardized infit *MSE* = 4.01) indicated that item 5 had more unexpected responses given person location and item location parameters. All other items adequately meet the fit requirements for Rasch analysis. In summary, the results from the dichotomous Rasch model analysis suggested that LLTM can be applied to MRT data to further explain item difficulty by item characteristics.

### 3.3. DIF Analysis for Gender

**Figure 2:**
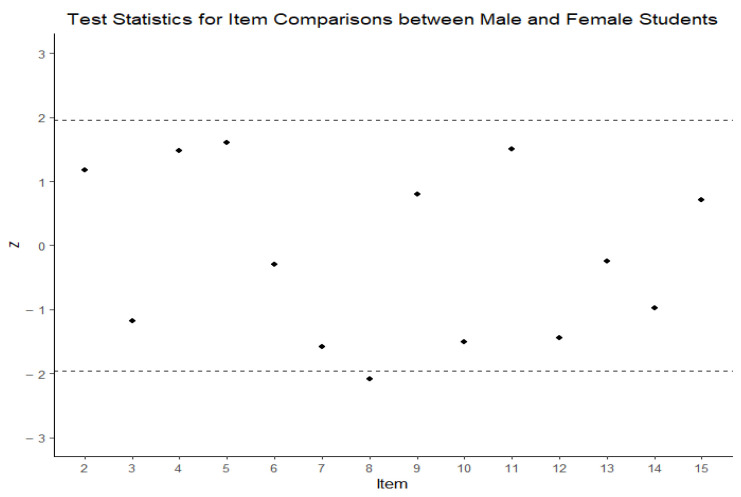
Scatterplot for standardized differences across gender for MRT items.

To investigate the fairness of the MRT items, this study initially considers standard DIF analysis. As suggested by Wright and Masters (1982), standardized differences in overall item difficulty parameters (across male and female students) are obtained and compared to the standard normal value at 0.05 level of significance (i.e., 1.96). The resulting overall item parameters from male and female groups along with their standardized differences and corresponding *z*-values are presented in Table 6. For better representations, standardized differences (estimated *z*-values) for all MRT items are plotted against boundaries for significant differences and presented in Figure 2. In Figure 2, the X axis represents the items, the Y axis represents the *z*-value, and boundaries for statistically significant differences are drawn at +1.96 and −1.96.

**Figure 3:**
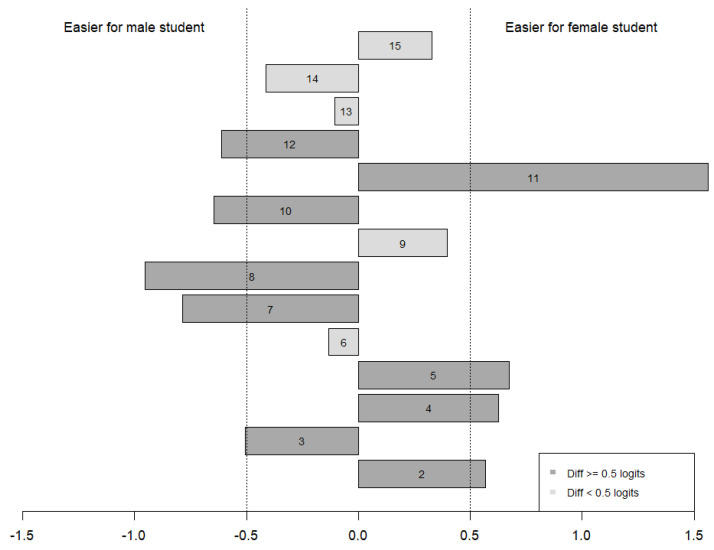
Bar plot representing logit difference in item difficulty for male and female students.

Reviewing results presented in both Table 6 and Figure 2, we can conclude that item 8 shows potential DIF (*z* = 2.09, *p* = 0.036). Figure 3 represents the logit difference of item difficulties for male and female students. Item 8 is relatively easier for male students compared to female students. Recognizing that the difference observed for item 8 was marginally significant, we kept the item in the instrument for further analysis. In practice, researchers sometimes interpret a logit difference value larger than 0.5 as evidence of a meaningful difference between groups but emphasize that these statistics are continuous values whose interpretation varies by context (Peabody and Wind 2019; Wright et al. 1976). Even though standardized differences suggested only one DIF item in the MRT instrument, we observed that some items (i.e., items 2, 4, 5, and 11) were potentially easier for female students, and some items (i.e., items 3, 7, 8, 10, and 12) were potentially easier for male students.

### 3.4. Results from LLTM Analysis

**Figure 4:**
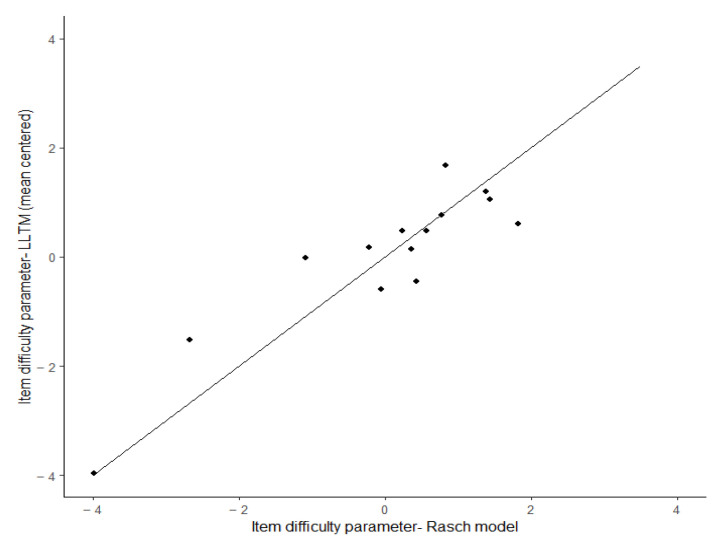
Scatterplot of estimated difficulty parameters from Rasch model and LLTM.

Based on the Rasch analysis, we removed item 5 from the analysis and re-estimated the Rasch model. To check overall model–data fit, we performed the LR test (Andersen 1973). The results showed adequate model–data fit for the Rasch model: $\chi^{2}=16.12,\;df=11,\;p=0.137\;$. In the dichotomous Rasch model, the −2log-likelihood was 1193.59, and for the LLTM model, the −2log-likelihood was 1317.28. Fit analysis for the LLTM showed a statistically significant difference of −2log-likelihoods between LLTM and Rasch model of 123.69 ($\chi^{2}\left(7\right),\;p<0.001$). The results indicated that the Rasch model had a better fit, and item characteristics in LLTM failed to capture all the variations in difficulty parameters. The findings are aligned with earlier studies that employed likelihood ratio tests to compare LLTM and Rasch models (Alexandrowicz 2011; Baghaei and Hohensinn 2017; Krell et al. 2021). This suggests that additional attributes may be needed to better capture sources of complexity (Baghaei and Hohensinn 2017; Baghaei and Ravand 2015). Furthermore, the difficulty parameters of the Rasch model and the LLTM were strongly and positively associated (*r* = 0.901), which indicates that $81.22\%\;\left(0.901^{2}\times 100\right)$ of the variance in the Rasch model’s item parameters can be explained by the six item characteristics considered in the LLTM model. As suggested by Baghaei and Hohensinn (2017), the *R*-square surpassed the threshold point of 0.76. Hence, the weight matrix for item characteristics provided meaningful information. In addition, a scatterplot of difficulty parameters from the Rasch model and LLTM (see Figure 4) suggests concurrence between LLTM and RM difficulty parameters.

The results from LLTM relevant to the item characteristics are presented in Table 7. The 95% confidence interval for each η parameter suggests that all item characteristics were significant (*p* < 0.05), which indicates that item characteristics considered in our analysis impacted item difficulty. Among all the characteristics, Ncubes (η=3.65) was most strongly associated with higher difficulty, suggesting that MRT items constructed with more cubes are more difficult to solve. Color also contributed significantly to the difficulty in MRT items. It is important to note that Ncubes and Color are likely to be confounded as most of the items possess similar characteristics or configurations. In addition, the complexity of the rotation η=1.50 was a significant predictor of item difficulty. On the other hand, item configuration (η=−1.07), occlusion (η=−0.62), and image structure (η=−0.59) appeared to reduce item difficulty. Results also suggest that structural image differences and homogenous item features increased item difficulty.

LLTM item difficulty parameters ($\delta^{L}$) are presented in Table 8. The LLTM difficulty parameters are estimated based on item characteristics parameters. As LLTM assumes the linear combinations of item characteristics parameters, $\delta^{L}$ estimates in Table 8 represent the sum of η estimates associated with each item. Conditioning on the effect of item characteristics, item 8 ($\delta^{L}=4.56$) was the most difficult item among MRT items, followed by item 7 ($\delta^{L}=4.08$). On the other hand, item 1 ($\delta^{L}=-1.07$) was the easiest item among all items considered in the MRT test. In our MRT instrument, item 1 was designed as an introductory question; we were therefore unsurprised by the low difficulty estimate for this item.

### 3.5. Results from Differential Component Functioning (DCF) Analysis via LLTM

To explore the DCF for gender via LLTM, we first separated the MRT response data based on gender. To perform the analysis, we considered males as the reference group and females as the focal group. As suggested by Fischer (1973), LLTM analysis can be performed if the dichotomous Rasch model fits the data adequately; hence, we fit the Rasch model separately to the male and female subsets. First, we explored the model–data fit of the dichotomous Rasch model for both male and female datasets using the LR test. The resulting measures were $\;\chi_{male}^{2}\left(11\right)=13.39,\;p>0.05$ and $\;\chi_{female}^{2}\left(10\right)=16.01,\;\;p>0.05$^1^. The results suggested that MRT item responses for both male and female groups fit the Rasch model adequately. We fit separate LLTM models for male and female students to assess potential variations in the effects of item characteristics within groups. To perform DCF analysis, we estimated the difference in effects of all item characteristics due to gender and performed a post hoc test employing Wald’s $\chi^{2}$ test. To perform Wald’s *χ*^2^ test, we applied the Bonferroni correction. For six simultaneous comparisons, the significance level was adjusted to 0.008 (α* = 0.05/6) based on a 0.05 overall level of significance. The η-estimates, along with estimated standard error and results from DCF analysis, are presented in Table 9.

The results from DCF analysis suggest that number of cubes and color have differential effects on item difficulty due to gender. For instance, the number of cubes (>8) tended to increase item difficulty more for male students than female students: $\chi^{2}\left(1\right)=11.11,\;p<0.001$. In addition, color in MRT objects also brought differential effects on item difficulty due to gender ($\chi^{2}\left(1\right)=11.04,\;p<0.001$). Note that we used high-contrast colors that are accessible to colorblind examinees. It is interesting to observe that color contributes to difficulty for male students, whereas color makes the items easier for female students. For other item characteristics, we did not observe any significant DCF related to gender.

## 4. Discussion

We examined the extent to which variation in item difficulty in MRT items can be explained by item-related characteristics and further explored whether the effects of item characteristics on item difficulty vary due to gender differences among students. To understand the contributions of MRT item characteristics on item difficulty, we employed explanatory IRT analysis, and the method enabled us to empirically assess the extent to which MRT item-related attributes contribute to item difficulties. The explanatory IRT approach, in explaining item difficulty using item-related attributes, is well discussed in the current literature and employed by several studies (Ivie and Embretson 2010; Krell et al. 2021; Shi et al. 2023). The LLTM analytical process applied in this study could be useful for education researchers to guide and evaluate item development procedures. This study further contributed to understanding test-takers’ gender as a potential source of variation in the effects of item characteristics on item difficulty. We employed Wald’s $\chi^{2}$ test to explore how the effects of item characteristics on item difficulty vary between gender groups. Exploring how effects of item characteristics may vary for gender may potentially be useful for exploring the effects of other demographic aspects on test development.

Our study aimed to provide details about how item characteristics may affect item difficulties in mental rotation assessments. We employed dichotomous specification of LLTM to assess how well the Q-matrix of item characteristics could account for item difficulty in the MRT instrument. Prior to implementing the LLTM analysis, we examined the psychometric properties of the MRT items. We employed both classical psychometric indicators and fit measures from the dichotomous Rasch model. Our results suggested that the items in the MRT instrument are consistent and exhibit adequate psychometric properties to implement LLTM. Furthermore, we performed DIF analysis as one perspective on fairness between groups for the MRT items and identified that one item in our MRT instrument had marginally significant DIF. By exploring the logit difference in item difficulties, we found that a few items were potentially easier for both male and female students.

To perform the LLTM analysis, we considered six item-level attributes. The Q-matrix for the item attributes was developed by experts associated with this study. From the LLTM analysis, we examined how MRT item attributes contributed to variance in item difficulties for students. The results suggested that item attributes play significant roles in explaining item difficulties in mental rotation tests. We found that some item attributes (i.e., Ncubes, color, rotation) significantly contributed to item difficulties in MRT items, indicating that items with an increased presence of these features may be more complex for students. Hence, item difficulties can be approximately pre-specified by controlling the presence and intensity of these features in MRT item constructions. The findings of this study are aligned with the results suggested by Lütke and Lange-Küttner (2015) and Ivie and Embretson (2010). However, our findings regarding the rotation complexity of this study do not align with the results concluded by Caissie et al. (2009). On the other hand, some features (i.e., occlusion, image structure, and item configuration) did not make an item more difficult but instead reduced item difficulty. For instance, we had dichotomized image structure based on two categories, i.e., structural image (0) and mirror image (1). Following the results obtained from LLTM, it can be concluded that structural images increase item difficulty. Furthermore, we used two categories to reflect item configuration (i.e., homogenous [1] and heterogenous [0]). Based on our results, it can be concluded that heterogeneity contributed more to item difficulty. These two findings imply that images lacking symmetry tend to be more difficult. In another study, Caissie et al. (2009) identified negative associations among difficulty parameters, occlusion, and configuration type. However, Doyle et al. (2016), reviewing Vandenberg and Kuse (1978) and Voyer and Hou (2006), concluded that occluded mental rotation items decrease item accuracy (i.e., increase difficulty). It is important to note that Vandenberg and Kuse (1978) employed a different item response structure (i.e., true/false) compared to this study.

We further explored how the contribution of item-related attributes to item difficulties varied between gender groups. Among six item-related attributes considered in this study, we identified two attributes that had statistically significant gender differences in their contribution to item difficulties: number of cubes and color. It is interesting to observe that the number of cubes contributed more to item difficulty for male students compared to female students. Furthermore, the introduction of differently colored cubes in an MRT item makes it more complex for male students^2^, while the color makes it easier to comprehend the item for female students. On the other hand, image structure, occlusion, rotation, and configuration had statistically similar contributions to item difficulty of MRT items for male and female students. The results of the present study are supported by some previous studies. For example, in a similar study, Voyer and Hou (2006) identified that image structure did not significantly impact item difficulty in MRT due to gender. Furthermore, Bors and Vigneau (2011) concluded that the degree of rotation in an MRT object (i.e., angular disparity) did not have any association with gender differences. Contrary to the results of the present study, few previous studies found that the magnitude of gender difference in MRT performance is prevalent for occluded items compared to non-occluded items (Bors and Vigneau 2011; Doyle and Voyer 2013; Doyle et al. 2016). However, the magnitude of gender difference in MRT performance for item configuration is sparsely studied.

This study has a few limitations. We acknowledge that the way we defined “gender” in this study was not explicitly clarified. Asking parents to self-identify their child’s “gender” could have led to confusion, as they may have interpreted this as either their biological sex or gender identity, depending on the respondent’s understanding.

While we effectively explained angular disparities qualitatively in our items, we found challenges in quantifying these rotations across three different dimensions in a way that was easily interpretable in the context of our research. Bartlett et al. (2024) correlated angles of rotation across each axis, along with skewed angles of rotation, with response patterns in their study. While they found significant effects of single-dimension rotations on response accuracy, no significant effect was found when correlating response accuracy with a multidimensional rotational measure. Future studies could explore methods to quantify multidimensional rotations in a way that is both mathematically rigorous and practically interpretable in the context of spatial reasoning and measurement.

For explaining item difficulties in the MRT, we only considered the main effects of item features and did not accommodate any interaction effects of item features. The interaction effects of item features may have additional implications in explaining item difficulties. Future studies may consider the interaction effects of item features to explain item difficulty by systematically developing items with different combinations of item characteristics. Furthermore, our results are provided based on a single mental rotation assessment. Subsequent studies may consider alternative mental rotation assessments encompassing diverse item types and samples with diverse and different backgrounds. In addition, we only considered the influence of gender on MRT item characteristics contributing to item difficulties. Future studies may consider other socio-economic factors to explore how item characteristics affect item difficulty for diverse groups of examinees. Lastly, this study was not pre-registered.

## 5. Conclusions

Mental rotation tests serve as a crucial tool for assessing spatial abilities, which involves manipulating spatial relationships between objects. Nevertheless, it is of paramount importance that the MRT effectively measure the abilities associated with mental rotation to aid the development of mental rotation abilities. Our study illustrated the application of the LLTM to assess a set of MRT items and examined the impact of various item characteristics on the difficulty of mental rotation assessment. All item characteristics included in the model significantly contributed to item difficulty. A higher number of cubes and the inclusion of color differences, structural differences, and homogenous figures increased difficulty, whereas occlusion was associated with a decrease in item difficulty. The number of cubes and color had the largest impacts on item difficulty, rotation and configuration had moderate impacts, and occlusion and image structure showed relatively smaller impacts. The presented results regarding item features may aid future item developers in systematically designing mental rotation items with varying difficulties. Furthermore, the methodology employed here may guide future researchers to identify different item attributes associated with item difficulties.

We performed DCF analysis that explored how the effect of item features varies due to gender differences employing Wald’s $\chi^{2}$ (Liao 2004) test. DCF is rarely discussed in the current literature and needs more attention. This approach in comparing the effects of item features allowed us to explore the variable nature of item features for clusters of test-takers with different demographic backgrounds. We identified two item features, the number of cubes and the color, that are perceived differently by male and female students, and these features have a variable impact on item difficulty due to gender. More specifically, these two characteristics increase difficulty for male participants more than female participants. This analytical approach provides additional insights about the cognitive reactions of students of different genders, allows us to measure how item features affect item difficulties across diverse population sub-groups, and provides insights about enhancing and ensuring equality of assessment in spatial reasoning tests relevant to mental rotations.

In conclusion, the present study emphasized the significance of effectively measuring spatial abilities through mental rotation assessments. This study identified key item features influencing item difficulty, offering valuable insights for future MRT instrument development. Furthermore, this study explored the nuanced impacts of gender on cognitive reactions to specific item features, contributing to a more inclusive and equitable assessment of mental rotation abilities.

## Figures and Tables

**Table 1:** Demographic distribution of students.

Demographic Factors	*N*	%
Gender		
Male	75	60.98%
Female	48	39.02%
Race		
Native American	8	6.50%
Asian	15	12.20%
Black/African American	4	3.25%
Hispanic	2	1.63%
White	90	73.17%
Other	4	3.25%

Note. *N* = 129; however, demographic information for six students is missing.

**Table 2:** Item-related characteristics for MRT items.

Item Characteristics	Description	Dichotomized Characteristics
Number of cubes (Ncubes)	Number of cubes in the rotated object.	1 if Ncubes > 8.6; 0 otherwise. See item 2 in Figure 1.
Color	If the distractors contain multiple colors.	1 if distractors contain multiple colors; 0 otherwise. See item 15 in Figure 1.
Image structure	Image structure is categorized into two categories, i.e., structural and mirror image. Mirror items are defined as items in which the distractors share the same segment of relationships as the target figure but are mirror images of it, whereas distractors are structurally different in structural formation.	0 if structural image; 1 if mirror image. See item 2 in Figure 1.
Occlusion	Occlusion refers to a situation where certain important portions of a three-dimensional object or figure are hidden or blocked from view as they are covered by other parts of the object.	1 if occlusion exists; 0 otherwise. See item 2 in Figure 1.
Rotation complexity	The rotation is simple if any distractors can be rotated with a single movement or complex if multidirectional movement is required.	1 if rotation is complex; 0 otherwise. See item 2 in Figure 1.
Configuration type	The MRT item configuration type is categorized in homogenous and heterogenous categories. The context refers to the categorization of objects or shapes based on a concept derived from the Vandenberg–Kuse items. These items are a set of objects or shapes that always consist of three segments or turns. An MRT item is homogenous if both ends of the shape have the same number of cubes and heterogenous if ends of the shape have a different number.	1 if the item configuration is homogenous; 0 if the item configuration is heterogenous. See item 11 in Figure 1.

Note. The item characteristic Ncubes was dichotomized using mean-split. We considered the cut-off point of 8.6, as the mean number of cubes for all the items was 8.6.

**Table 3:** Q-matrix developed for item characteristics/components.

Item	Ncubes	Color	Image Structure	Occlusion	Rotation	Configuration
1	0	0	0	0	0	1
2	1	0	1	1	1	1
3	1	0	1	1	0	0
4	1	0	1	0	0	0
5	0	0	0	1	0	0
6	1	0	0	0	0	0
7	1	0	0	0	1	1
8	1	0	1	0	1	0
9	1	0	1	0	1	1
10	1	0	0	1	0	0
11	0	1	0	0	0	1
12	0	1	1	0	1	1
13	0	1	1	0	1	0
14	0	1	1	0	1	0
15	0	1	0	0	1	0

**Table 4:** Summary of MRT item statistics.

Item	Proportion Correct (*M*)	*SD*	Corrected Item-Total Correlation
1	0.98	0.15	0.16
2	0.74	0.44	0.46
3	0.47	0.50	0.53
4	0.59	0.49	0.43
5	0.53	0.50	0.16
6	0.40	0.49	0.38
7	0.30	0.46	0.41
8	0.40	0.49	0.40
9	0.24	0.43	0.39
10	0.48	0.50	0.52
11	0.92	0.27	0.29
12	0.56	0.50	0.46
13	0.44	0.50	0.54
14	0.50	0.50	0.45
15	0.29	0.46	0.46

Note: sample size, *n* = 129; *M* = mean, *SD* = standard deviation; corrected item-total correlation = correlation with specific item with the total score of all other items in the score.

**Table 5:** Item parameter estimates from dichotomous Rasch model.

Item	δ	*SE*	Lower CI	Upper CI	Outfit *MSE*	Infit *MSE*	Outfit *z*	Infit *z*
1	−3.98	0.56	2.88	5.08	0.32	0.79	−0.50	−0.36
2	−1.08	0.22	0.65	1.50	0.61	0.80	−1.85	−2.07
3	0.44	0.20	−0.84	−0.04	0.82	0.87	−1.17	−1.44
4	−0.21	0.20	−0.18	0.60	0.84	0.95	−1.02	−0.59
5	0.11	0.20	−0.51	0.28	1.38	1.39	2.30	4.01
6	0.78	0.21	−1.19	−0.38	1.28	1.07	1.51	0.76
7	1.39	0.22	−1.83	−0.95	1.08	1.03	0.39	0.31
8	0.83	0.21	−1.23	−0.42	1.23	1.05	1.27	0.52
9	1.83	0.24	−2.30	−1.36	1.58	1.04	1.73	0.36
10	0.36	0.20	−0.75	0.04	0.87	0.87	−0.86	−1.42
11	−2.67	0.33	2.02	3.32	0.41	0.72	−1.20	−1.28
12	−0.05	0.20	−0.34	0.44	0.88	0.93	−0.73	−0.86
13	0.57	0.20	−0.97	−0.17	0.78	0.86	−1.45	−1.49
14	0.24	0.20	−0.63	0.16	0.98	0.96	−0.11	−0.40
15	1.44	0.23	−1.89	−1.00	0.83	1.00	−0.66	0.00
M	0.00	0.24	−0.47	0.47	0.93	0.96	−0.16	−0.26
SD	1.55	0.09	1.40	1.70	0.34	0.16	1.29	1.46

Note: *SE* = standard error, CI = confidence interval at 95% confidence, *MSE* = mean square error.

**Table 6:** Results from DIF analysis for MRT response data across gender.

Item	$\delta_{m}$	$SE_{m}$	$\delta_{F}$	$SE_{F}$	*z*	*p*
2	−1.20	0.28	−1.77	0.39	1.18	0.238
3	−0.02	0.26	0.49	0.34	−1.18	0.236
4	−0.23	0.26	−0.86	0.34	1.48	0.139
5	0.26	0.26	−0.41	0.33	1.61	0.107
6	0.48	0.26	0.61	0.35	−0.30	0.761
7	0.85	0.27	1.63	0.42	−1.58	0.113
8	0.19	0.26	1.14	0.38	−2.09	0.036 *
9	1.69	0.30	1.30	0.39	0.80	0.421
10	−0.16	0.26	0.49	0.34	−1.51	0.131
11	−2.71	0.42	−4.27	0.96	1.50	0.134
12	−0.59	0.26	0.03	0.33	−1.45	0.146
13	0.26	0.26	0.37	0.34	−0.25	0.801
14	−0.16	0.26	0.25	0.34	−0.98	0.328
15	1.33	0.28	1.00	0.37	0.71	0.479

Note: $\delta_{m}$ and $\delta_{F}$ are item difficulty parameter estimates for male and female students, respectively; $SE_{m}$ and $SE_{F}$ are standard error for item difficulty parameters for male and female students; * *p* < 0.05.

**Table 7:** Calibration of item characteristics using LLTM with 95% CI.

Item Characteristics	η	*SE*	Lower CI	Upper CI
Ncubes	3.65	0.61	2.44	4.85
Color	2.45	0.62	1.23	3.66
Image structure	−0.59	0.13	−0.85	−0.32
Occlusion	−0.62	0.16	−0.92	−0.31
Rotation	1.50	0.16	1.18	1.81
Configuration	−1.07	0.13	−1.33	−0.80

Note: *SE* = standard error; CI = confidence interval at 95% level of confidence; see Table 1 for item characteristic definitions.

**Table 8:** Estimated item difficulty parameters from LLTM.

Item	$\delta^{L}$	*SE*	Lower CI	Upper CI
1	−1.07	0.13	−1.32	−0.82
2	2.87	0.63	1.64	4.10
3	2.44	0.62	1.22	3.66
4	3.06	0.62	1.84	4.28
6	3.65	0.61	2.45	4.85
7	4.08	0.63	2.85	5.31
8	4.56	0.61	3.36	5.76
9	3.49	0.63	2.26	4.72
10	3.03	0.61	1.83	4.23
11	1.37	0.66	0.08	2.66
12	2.29	0.63	1.06	3.52
13	3.36	0.60	2.18	4.54
14	3.36	0.60	2.18	4.54
15	3.94	0.61	2.74	5.14

Note: *SE* = standard error, CI = confidence interval at 95% level of confidence.

**Table 9:** Difference in effects of item characteristics across gender via LLTM.

Item Characteristics	$\eta_{ref}$	$SE_{\eta_{ref}}$	$\eta_{focal}$	$SE_{\eta_{focal}}$	*Deviance in Effect*	$\chi_{\left(1\right)}^{2}$	*p*
Ncubes	2.99	0.64	0.48	0.40	2.51 ***	11.11	<0.001
Color	1.88	0.65	−0.79	0.47	2.66 ***	11.04	<0.001
Image structure	−0.53	0.17	−0.67	0.22	0.14	0.25	0.615
Occlusion	−0.68	0.20	−0.51	0.25	−0.17	0.27	0.600
Rotation	1.31	0.20	1.75	0.27	−0.44	1.68	0.195
Configuration	−0.93	0.17	−1.32	0.23	0.39	1.86	0.173

Note: $\eta_{ref}$ and $\eta_{focal}$ represents estimated effects of item characteristics. ref: male students, focal: female students. *** *p* < 0.001.

## Data Availability

The mental rotation test response data employed in this study can be accessed here: https://osf.io/wa53j (accessed on 21 December 2024).

## Notes

1 All examinees in female group responded to item 1 correctly. Hence, item 1 was discarded from the female data set.

2 Note that the two colors used in these items were selected to maximize the contrast for students with color blindness, so that would not be a factor.
